# Predicting poor outcomes in children aged 1–12 with respiratory tract infections: A systematic review

**DOI:** 10.1371/journal.pone.0249533

**Published:** 2021-04-19

**Authors:** George Edwards, Louise Newbould, Charlotte Nesbitt, Miranda Rogers, Rebecca L. Morris, Alastair D. Hay, Stephen M. Campbell, Gail Hayward

**Affiliations:** 1 Nuffield Department of Primary Care Health Sciences, University of Oxford, Oxford, United Kingdom; 2 Social Policy Research Unit, University of York, York, United Kingdom; 3 Division of Population Health, NIHR Greater Manchester Patient Safety Translational Research Centre, School of Health Sciences, University of Manchester, Manchester, United Kingdom; 4 Centre for Academic Primary Care, Bristol Medical School: Population Health Sciences, University of Bristol, Bristol, United Kingdom; University of Children’s Hospital of Bern, SWITZERLAND

## Abstract

**Background:**

Demand for NHS services is high and rising. In children respiratory tract infections (RTI) are the most common reason for consultation with primary care. Understanding which features are associated with good and poor prognosis with RTI will help develop interventions to support parents manage illness.

**Aim:**

To identify symptoms, signs, and investigation results associated with good and poor prognosis, and clinical decision making in children aged 1–12 years with RTI symptoms, at home and presenting to ambulatory care.

**Design and setting:**

Systematic literature review.

**Methods:**

We searched MEDLINE, EMBASE, Cinahl, Web of Science and the Cochrane database of systematic reviews for studies of children aged 1 to 12 years with a RTI or related condition reporting symptoms, signs and investigation results associated with prognostic outcomes. Quality was assessed using the QUIPS tool.

**Results:**

We included 27 studies which included 34802 children and measured 192 factors. Nine studies explored future outcomes and the remainder explored clinical management from the initial consultation with the health services. None were conducted in a home setting. Respiratory signs, vomiting, fever, dehydration and tachycardia at the initial contact were associated with future hospitalisation. Little evidence was available for other outcomes.

**Conclusion:**

Some evidence is available to clinicians to stratify risk of, future hospitalisation, but not of other prognostic outcomes. There is little evidence available to parents to identify children at risk of poor prognosis. Research is needed into whether poor prognosis can be predicted by parents in the home.

## Introduction

Use of the National Health Service (NHS) is high and rising [1, 2]. Respiratory tract infection (RTI) is a common illness amongst children under the age of 12 [3] and is the most common paediatric presentation managed by primary care clinicians [4, 5]. Most RTIs are self-limiting illnesses, albeit with longer courses than expected by both clinicians and parents [6–8], and are rarely serious enough to require hospital admission [9]. Despite this, they can be a significant cause of anxiety for parents [10].

There is uncertainty from both parents and clinicians about how to identify children with RTIs who are at risk of poor prognosis [11]. Parents often take their children to visit the health services due to uncertainty about their condition [12, 13] and to ‘rule out’ a potential health threat [13] which they may be unable to recognise themselves [14]. Clinicians express similar concerns in predicting prognosis of intermediate severity RTIs [15] and may prescribe antibiotics to mitigate deterioration in these cases. Antibiotic prescription is a common outcome from RTI consultations [16, 17] and more than 60% of all inappropriate antibiotic prescriptions issued in primary care are for RTI and ear, nose and throat conditions including sore throat and cough [18]. This can lead to antibiotic resistance [19].

One approach to reducing demand for consultations amongst children with RTIs is to develop guidance for parents on when it is most appropriate to care for children at home or visit a pharmacy and when to seek healthcare advice for paediatric RTI. A systematic review found that a patient education programmes which engage parents and children may reduce consulting rates by 13–40% [20]. However, the evidence for predicting prognosis in paediatric RTI is unclear. Therefore we aimed to systematically review the literature on the association between symptoms, signs, and investigations and clinical decision making and risk of poor future outcomes in children aged between 1 and 12.

## Methods

Our review protocol was registered with PROSPERO: CRD42019122487

### Search

We developed a search strategy using an iterative process based on recommendations from an information specialist. We searched Medline, Embase, Cinahl, Cochrane database of systematic reviews, and the Web of Science for studies with key words in four main themes: the age of the target patients, the conditions of interest, target settings, and key outcomes (see S1 Table in S1 File for Medline search terms). We limited the search to studies in English. The search was carried out on the 23^rd^ January 2019 and was re-run on 10^th^ August 2020.

### Eligibility

#### Participants

Children between the ages of 1 and 12 (inclusive). We excluded studies including only children younger than one as it was considered that a GP would be more likely to offer an assessment to this age group. Studies of children over the age of 12 only were not included as children beyond this age have a greater role in deciding whether to consult. We excluded studies of children with comorbidities other than obesity and asthma.

#### Conditions

Undifferentiated RTI, common cold, influenza, cough (≤28 days), respiratory wheeze, breathing difficulties, otitis media, ear ache, croup, bronchiolitis, and pneumonia. We excluded studies of asthma, malaria, cystic fibrosis, tuberculosis, acute cough lasting longer than 28 days, and any infectious agent not related to the respiratory tract.

#### Prognostic factors

Symptoms, clinical signs, and investigation results available to primary care. We classified symptoms as anything apparent to parents (or older children), such as cough, and clinical signs as evidence available to clinicians on physical examination, such as crackles on auscultation. We included investigation results if they were potentially available to primary care clinicians, such as point-of-care tests or oxygen saturation tests. We excluded studies investigating the prognostic value of clinical prediction rules unless results were reported separately for individual factors.

#### Outcomes

To account for poor outcomes we include five measures of poor prognosis: death, hospitalisation, antibiotic prescriptions, prolonged or deteriorating symptoms, and re-consultation, and two clinical decisions: immediate hospitalisation and antibiotic prescription. We excluded studies which did not report any of these outcomes.

#### Settings

Primary care, ambulatory care, emergency department, and the home setting. We excluded any study not defined as a World Bank high income country [21] as the disease spectrum is likely to be different.

#### Type of study

Prospective or retrospective cohort, case-control, cross-sectional studies, and systematic reviews. We excluded all other study types.

### Selection of studies

The Cochrane Collaboration Covidence platform was used for study screening. Two authors (GE, LN, MR, or CN) screened each study according to pre specified inclusion and exclusion criteria and we resolved disagreements by discussion with a third reviewer (GH). We screened titles and abstracts initially and obtained full texts for potentially relevant studies. We hand searched reference lists of all included studies and relevant systematic reviews for included studies.

### Data extraction

For each included study, two authors (GE, LN, MR, CN, or RM) performed data extraction independently using a standardised and piloted data extraction form. We extracted general study information, study characteristics, participants, prognostic factors and outcome measures, analysis techniques, and results. We extracted multivariate odds ratios as a measure of the effect size where possible.

### Risk of bias assessment

For each included study, two authors (GE, LN, MR, CN, or RM) assessed the risk of bias using the QUIPS tool [22]. One author (GE) checked for consistency across all studies.

### Analysis

Due to study heterogeneity we were unable to perform meta-analyses and have reported outcomes narratively.

## Results

### Summary of included studies

**Fig 1:**
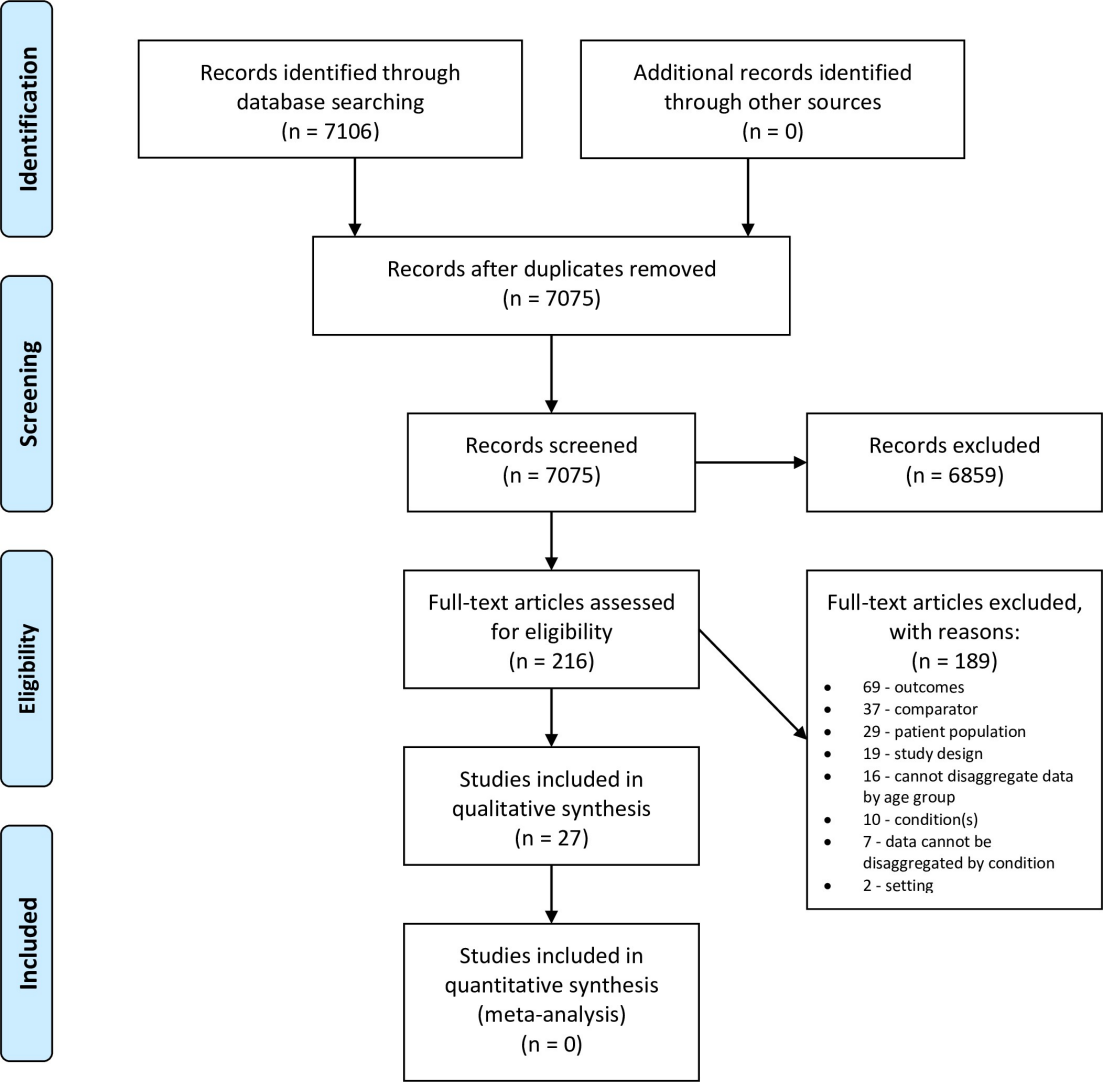
PRISMA flow diagram.

The database search identified 7106 articles (Fig 1). Of these we selected 216 for a full text review and included 27. These studies included 34221 children and analysed 192 different prognostic factors.

Studies included unselected children with RTI (n = 11), and children with clinical diagnoses of bronchiolitis (6), influenza (5), acute otitis media (1), community acquired pneumonia (2), croup (1), and peritonsillar abscess (1). Seven studies were performed in the UK, four in the USA, four did not specify, and one was conducted in 12 different countries. The remaining studies were conducted in 12 different high income countries.

There were no studies conducted in a home setting. Settings included: emergency department (16), primary care (7), hospital inpatient ward (2), mixed settings (1), and ambulatory care (1). Study outcomes were death (3), future hospitalisation (2), prolonged symptoms (3), re-consultation (2), future antibiotic prescription (1), hospitalisation from the initial consultation (15), and antibiotic prescription at the initial consultation (6). Full characteristics of included studies are detailed in Table 1.

**Table 1:** Included study summary.

Study	Population (n, mean or median age in years, IQR or SD of age in years % under 1% over 12)*	Presenting Condition	Study design	Country	Setting	Primary outcome	Additional Outcomes
Ambroggio 2018 [23]	n = 3016,	Community acquired pneumonia	Retrospective cohort study	Not specified	ED	Pneumonia-related ED revisit (within 48 hours or 30 days)	Hospital admission within 30 days with a diagnosis code for pneumonia
	median 3.0,						
	IQR 1.7,						
Butler 2005 [7]	n = 240	Probable viral acute upper respiratory tract illness	Secondary analysis of a randomised controlled trial	UK	Situation of usual care	Variables associated with scores on the Canadian Respiratory Illness and Flu Scale (CARFIS [24]) after 7 days	Re-consultation; Antibiotic prescription after 7 days
	mean 5.2 years						
	standard deviation						
	(SD) 3.39						
	All <12 years						
Dalziel 2013 [25]	n = 265 cases	Influenza like illness	Retrospective case-control	12 different countries	ED	Hospitalisation to intensive care for assisted ventilation and or inotropic/vasopressor support	Death
	mean 6.6 years						
	SD 4.7						
Dubnov-Raz 2011 [26]	n = 73	Laboratory confirmed H1N1 Influenza	Prospective cohort study	Israel	Tertiary care hospital	Hospitalisation for at least 24 hours	Death
	mean 8.9 years						
	SD 5.2						
Hay 2003 [6]	n = 256	Cough ≤28 days	Prospective cohort study	UK	Primary Care	Cough duration	
	median 2						
	snterquartile range (IQR) 6.8–35.0						
	All <12 years						
Hay 2016 [9]	n = 8394	Cough ≤28 days and other respiratory tract infection (RTI) symptoms	Prospective cohort study	UK	Primary Care	Hospital admission for RTI within 30 days	
	median 3						
	IQR 1–6						
Venkatesan 2015 [27]	n = 159 (target population 0–16 years)	Influenza like illness	Open cohort study—30 day follow up	UK	Primary Care	Hospital admission within 24 hours of assessment in general practice	Death within 30 days; Antibiotic prescription
Walsh 2004 [28]	n = 132	Bronchiolitis	Retrospective chart review	Ireland	ED	Hospitalisation within 24 hours of initial consultation	
	mean 6.1 months						
	(range 0.27–21.9 months)						
Wensaas 2018 [29]	n = 2398	Acute cough ≤28 days and other RTI symptoms	Prospective cohort study	UK	Primary Care	Prediction of one of five symptom trajectories	
	mean 4.1 years						
Yang 2017 [30]	n = 196	Croup	Prospective cohort study	Taiwan	Paediatric ED	Admission to paediatric observation unit, in-patient ward, or ICU	Re-consultation
	mean 2.1 years						
	SD 1.8						
	19.8% aged <1						
Ahmed 2010 [31]	n = 6134 (target range 0–18	Respiratory Illness	Retrospective cross-sectional chart review	Not specified	Family practitioner office, paediatrician office, ED	Antibiotic prescription at the initial contact	
Alzaharani 2018 [32]	n = 4013 (target range 6 months to 18 years)	Upper Respiratory Infection	Cross sectional study of publically available data	Not specified	Ambulatory Care	Broad spectrum prescription at the initial contact	
Bender 2009 [33]	n = 1230 (target range 0–18)	Laboratory confirmed influenza	Retrospective cohort study	USA	ED	Admission to the paediatric ward, a 24-hour observation unit, or intensive care at the initial contact.	
Blacklock 2011 [34]	n = 700	Suspected acute infection	Prospective diagnostic cohort study	UK	Paediatric Assessment Unit	Serious respiratory infection’ (consolidation on chest x-ray, clinical diagnosis of lower RTI, or RTI requiring hospitalisation and one inpatient treatment),	Other foci of serious infection; Minor or no infection
	mean 3.5 years						
	SD 3.2						
	20.2%						
	All <12 years						
Brown 2003 [35]	n = 679	Bronchiolitis	Retrospective chart review	Not specified	ED	Hospitalisation through the ED at the initial contact with a ED diagnosis consistent with bronchiolitis and a hospital discharge of bronchiolitis	
	median 7 months						
	IQR 4–11 months						
	All <12 years						
Ciofi Degli Atti 2006 [36]	n = 2151	Respiratory tract infections	Cross sectional survey	Italy	Primary Care	Antibiotic prescription at the initial contact	
	mean 4.2 years						
Florin 2020 [37]	n = 477	Community-acquired pneumonia	Prospective cohort study	USA	ED	Hospitalisation at initial consultation or within 7 days	
	mean 5.6						
	SD 4.6						
Garcia 2015 [38]	n = 695	Laboratory confirmed H1N1 Influenza	Retrospective cross sectional case review	USA	Patients on hospital record	Admission to hospital ward or to intensive care	
	(target range 0–18 years)						
Gotta 2017 [39]	n = 169, *(target 1 month—18 year)	Lower respiratory tract infection	Secondary analysis of a randomised controlled trial	Switzerland	Paediatric ED	Antibiotic prescription at the initial contact	
Millar 2007 [40]	n = 257 (included range 1–17 years)	Peritonsillar Abscess	Retrospective chart review	Canada	ED	Admission to hospital on initial presentation	
Pailhous 2015 [41]	n = 348	Acute bronchiolitis	Prospective cohort study	France	ED	Admission to hospital on initial presentation	
	median 18 weeks						
	(included range 0–100 weeks)						
	All <12 years						
Parker 2009 [42]	n = 312 (target range 2–23 months)	Bronchiolitis	Secondary analysis of a prospective cohort study	Canada	ED	Major medical intervention (oxygen administration for 30 minutes or more for saturation <90% in room air, IV fluid bolus of 20ml/kg or more, treatment for apnoea, admission to the Critical Care Unit).	
	All <12 years						
Pruikkonen 2014 [43]	539, (target 6 months-16 years)	Wheeze during respiratory infection	Retrospective chart review	Finland	ED	Hospitalisation for at least 48 hours or intensive care admission	
Rebnord 2017 [44]	n = 401	Fever and/or respiratory symptoms	Secondary analysis of randomised controlled trial	Norway	Four out-of-hours general practice services and one paediatric emergency clinic	Antibiotic prescription at the initial contact	Hospital admission from the initial contact
	mean 2.3 (median 1.6)						
	All <12 years						
Smith 2010 [45]	n = 256 (target range 6 months-10 years)	Acute otitis media	Observational cohort study	UK	Primary Care	Antibiotic prescribed by general practitioner (timeframe unclear)	
Voets 2006 [46]	n = 405	Bronchiolitis	Prospective cohort study	Belgium	ED	Admission to hospital on initial presentation	
	average 9.4 months						
	All <12 years						
Yusuf 2012 [47]	n = 325 (target range 0–2 years)	Bronchiolitis	Retrospective cohort study of all patients younger than 2 with bronchiolitis who were monitored in Texas Children’s hospital	USA	Emergency Department Observation Unit	Admission to hospital from the paediatric observation unit.	
	78.8%						
	All <12 years						

**Grey shading** indicates studies where at least one outcome was assessed at a timepoint beyond the initial contact where symptoms were documented (eg hospitalisation within 30 days, symptom duration).

*where data is absent from this column it is unavailable. ED = Emergency Department.

### Risk of bias of included studies

Full QUIPS assessment is available in Table 2. We found a high risk of confounding in 9/27 studies with 11/27 at moderate risk. Nine of the studies had a retrospective design which makes controlling for confounding variables, such as other relevant signs and symptoms, challenging. However, even in the prospective studies there was limited reporting or discussion of potential confounding variables.

**Table 2:** Risk of bias assessment summary.

Study	Study Participation	Study Attrition	Prognostic Factor Measurement	Outcome Measurement	Study Confounding	Statistical Analysis and reporting
Ahmed 2010 [31]	Low	Low	Low	Low	Medium	Low
Alzaharani 2018 [32]	Low	Low	Medium	Low	Medium	Low
Ambroggio 2018 [23]	Low	Medium	Low	Low	Medium	Low
Bender 2009 [33]	Low	Low	Low	Low	High	Low
Blacklock 2011 [34]	Low	Medium	Low	Low	High	Low
Brown 2003 [35]	Medium	Low	Medium	Low	High	Low
Butler 2005 [7]	Medium	High	Medium	Low	High	Medium
Ciofi Degli Atti 2006 [36]	Medium	Medium	Low	Low	Medium	Low
Dalziel 2013 [25]	Medium	Low	Medium	Low	High	Medium
Dubnov-Raz 2011 [26]	Low	Low	Low	Low	High	Low
Florin 2020 [37]	Medium	High	Medium	Low	High	Low
Garcia 2015 [38]	Low	Low	Low	Low	Low	Low
Gotta 2017 [39]	Low	Low	Medium	High	Medium	Low
Hay 2003 [6]	Low	Medium	Low	Low	Low	Low
Hay 2016 [9]	Low	Low	Low	Low	Low	Low
Millar 2007 [40]	Low	Low	Medium	Medium	Medium	Medium
Pailhouse 2015 [41]	Low	Medium	Medium	Low	High	Medium
Parker 2009 [42]	Medium	Low	Low	Low	Medium	Low
Pruikkonen 2014 [43]	Low	Low	Medium	Low	Low	Low
Rebnord 2017 [44]	Medium	High	Low	Low	High	Low
Smith 2010 [45]	Medium	High	High		Medium	High
Venkatesan 2015 [27]	High	High	High	Low	Low	Low
Voets 2006 [46]	Medium	Low	Low	Low	Medium	Medium
Walsh 2004 [28]	Low	Low	Medium	Low	High	Medium
Wensaas 2018 [29]	Low	Medium	Low	Low	Medium	Low
Yang 2017 [30]	Medium	Low	Medium	Low	High	High
Yusuf 2012 [47]	Low	Low	Low	Low	Medium	Low

In 11/27 studies we found a moderate risk of bias in prognostic factor measurement with 1 study at high risk. Key issues were poor reporting of the method of recording and measurement of prognostic factors and a lack of pre-specification of cut offs for continuous variables (e.g. fever) [7, 35, 39]

A final concern related to statistical reporting and analysis. We found a moderate risk of bias in 6/27 studies and a high risk of bias in 2/27. Key issues arising in this domain were a lack of available data to assess the adequacy of the analysis [40, 45] and a lack of justification for the statistical methods and models used [30].

### Factors associated with poor or good prognosis

192 different prognostic factors were explored (see S2 Table in S1 File for full details of prognostic factors in each study). Each study measured prognostic factors in one cross-section; no studies measured prognostic factors at multiple time points.

Study outcomes are reported in two groups: prognostic (death, future hospitalisation or antibiotic prescription, prolonged symptoms, and re-consultation), and clinical decision making (immediate hospitalisation or antibiotic prescription).

#### Prognostic

*Death*. Three studies, including 497 patients with suspected [27] or laboratory confirmed [25, 26] influenza, reported on deaths. Only one study analysed the predictive value of patient features; no patient signs, symptoms, or investigation results were associated with death [25]. One study did not observe any deaths [26], and the final study lacked sufficient data for analysis [27].

*Hospitalisation within 30 days*. One study investigated the association of symptoms, signs and investigations with hospitalisation within 30 days from initial contact with primary care for acute cough or respiratory tract infection in 8394 children aged 3 months to 16 years [9]. Of 40 relevant prognostic factors analysed, four were associated with hospitalisation within 30 days of the initial consultation after multivariate analysis: moderate-to-severe vomiting (2.56 95% CI 1.54–4.31), parent reported severe fever or a temperature of ≥37.8°C (1.99 95% CI 1.22–3.25), wheeze (2.16 95% CI 1.28–3.60), and intercostal and subcostal recession (3.82 95% CI 2.23–6.62). In this study, the baseline risk of hospitalisation within 30 days was 0.9%. Although recession was associated with the largest odds ratio for the study outcome, only 7% of patients presenting with recession were hospitalised within 30 days. We found a low risk of bias across 5/6 domains for this study, and a medium risk in the final domain (study attrition).

*Hospitalisation within 24 hours*. One study assessed 19 relevant prognostic factors in a population of 132 children aged 0–2 years presenting to ED with a subsequent diagnosis of bronchiolitis [28]. Of these three were associated with modest increases in odds of hospitalisation: increased work of breathing (3.39 95% CI 1.29–8.92 p = 0.013), dehydration (2.54 95% CI 1.34–4.82 p = 0.004), and heart rate above 97^th^ centile for each age (3.78 95% CI 1.05–13.57 p = 0.041). For this study, we found a high risk of bias due to confounding, and a medium risk of bias due to prognostic factor measurement, and statistical analysis and reporting. Potentially confounding variables were not considered or controlled for, and some details regarding prognostic factors and their statistical significant was lacking.

*Prolonged or deteriorating symptoms*. We included three studies exploring the association between symptoms and prolonged duration or deterioration [6, 7, 29] in 2945 children consulting with a suspected RTI.

One study identified five trajectories of recovery from acute cough [29] but baseline symptoms were unable to identify the trajectory of any given child. Parent reported severe cough in the 24 hours before exam was associated more severe and longer lasting cough trajectories. We had no major concerns regarding the risk of bias for this study and found a medium risk of bias in 2/6 domains, and a low risk in 4/6. In a second study [6] of pre-school children with a cough of less than 28 days and without asthma there was no difference in cough length between patients with or without abnormal chest signs, fever, or tachypnoea. We had few concerns regarding the risk of bias for this study and found a medium risk of bias in 1/6 domains, and a low risk in 5/6.

One study [7] of 290 children consulting a site of usual care with suspected acute viral upper respiratory tract infection recorded 18 individual severity score items (CARIFS items [24]) scored by parents or carers on the day of the initial consultation and six clinician recorded symptoms. Of these, patients with parent or carer reported fever and low energy/tiredness were more likely to have a higher parent reported CARIFS score (indicating worse symptoms) after seven days. These two variables explained 15% of the variation in CARIFS score after seven days. Patients with a clinician recorded cough at the initial consultation were also more likely to have higher CARIFs score after seven days. We found a moderate risk of confounding in three domains (study participation, prognostic factor measurement, and statistical analysis and reporting) and a high risk in two (confounding and study attrition).

*Re-consultation*. We identified two studies including 240 children with suspected acute viral upper respiratory tract infection and 3016 children with community acquired pneumonia [7, 23]. In primary care, Butler et al found was no association between 18 individual severity score items (CARIFS items [24]) and six clinician identified factors recorded on the day of the initial consultation and re-consultation [7]. Patients discharged from ED with a diagnosis of community acquired pneumonia patients were more likely to re-consult if they had consulted with fever at the index visit (OR 2.24 95% CI 1.29–3.90) [23].

*Antibiotics within 14 days*. Butler et al found that none of 18 parent reported CARIFS items on the day of the initial consultation [24] or six clinician identified symptoms were associated with antibiotic prescription within 14 days in children not receiving a prescription at their initial consultation.

#### Factors associated with measures of raised clinical concern

*Hospitalisation*. 15 studies examined associations between prognostic factors and hospitalisation from the initial contact [23, 25–27, 30, 33–35, 37, 38, 40–44, 46, 47]. There was a range of different definitions of ‘hospitalisation’ (see S1 Table in S1 File). Factors with significant associations for children with unspecified RTI, influenza, bronchiolitis and other conditions are reported separately in Table 3. In general we had concerns about the risk of bias in these studies; we found several to have a high risk of bias due to confounding linked with the retrospective design of the studies (Table 2).

**Table 3:** Factors associated with hospitalisation from the initial contact.

*Setting / Clinical condition / Number of children (n =)*	*Likelihood of being hospitalised (associated factors)*		*Discrepant findings between studies and possible explanations (in italics)*
	*Increased*	*Reduced*	
**Settings**: Primary Care [27, 44]	Respiratory distress on clinical examination [34]; respiratory rate as a continuous variable [44]; signs on auscultation [44] pallor [34]; temperature of ≥39 ^o^C (although in the same study a threshold of ≥38°C was not significant) [34]	Findings on ear examination [44]	**Oxygen saturation:** Associated with hospitalisation at two thresholds, 90–95% [44], and <94% [34], but not at one further threshold, <90%.
ED [25, 43]			
Paediatric assessment unit [34]			*One study was in an acute settings* [34], *the other in a primary care setting* [44]
**Clinical condition:** RTI			
*n =* 1640			
**Setting:** Emergency Department	Abnormal lung auscultation [26]; abnormal x-ray or radiologic evidence of pneumonia [26, 33]; chest wall retraction [25]; signs of dehydration [25]; oxygen saturation <93% [25]; fatigue [38]; tachycardia [38]; laboratory confirmed influenza B [33]; respiratory distress [33]; shortness of breath or dyspnoea [26, 38]	Headache [38]	**Myalgia and Feve**r: Associated with reduced odds of hospitalisation in one study [38] but not a second study [26]
		Congestion [38]	
**Clinical condition:** Influenza		Chills [38]; respiratory exhaustion [27]	*There were no differences in setting or the ages of children between these studies*. *The study finding a significant relationship included a larger cohort*.
*n =* 2422			
**Setting:** Emergency Department	Food intake of < 50% of the usual amount [41]; decreased hydration [28, 42]; respiratory rate >60/minute [41, 42]; an accessory muscle score of ≥6/9; tachycardia above the 97^th^ percentile for age		**Increased work of breathing:** Associated with increased odds of hospitalisation in one study [28] but not another [47].
Observation Unit			
			**Lower oxygen saturation:** Associated with an increased odds of hospitalisation at four thresholds: <95 [46], ≤94 [41], <93% [47] and ≤92% [42]. One study found no association between oxygen saturation as a continuous variable and hospitalisation [35].
**Clinical condition:** Bronchiolitis			
*n =* 2201			
			*The setting*, *age group*, *and size in these studies is similar*. *We found the study with no association to have a high risk of confounding*.
**Setting:** Emergency Department	CRP [37]; procalcitonin [37]		No discrepant findings
**Clinical condition:** Community	(Increased odds of hospitalisation per doubling of biomarker value)		
acquired pneumonia			
*n =* 477			

A grey box indicates that no associations were found.

*Antibiotics*. Six studies analysed the association between symptoms, signs, and investigation and antibiotic prescription at the initial consultation. There were five studies analysing associations with antibiotic prescription at the initial contact in 12868 children consulting with a suspected respiratory tract infection [31, 32, 36, 39, 44] and one study of 256 children presenting with otitis media in routine clinical practice [45]. Significant associations are reported in Table 4.

**Table 4:** Factors associated with an antibiotic prescription at the initial contact.

*Setting / Clinical condition / Number of children (n =)*	*Likelihood of being prescribed antibiotics (associated factors)*		*Discrepant findings between studies and possible explanations (in italics)*
	*Increased*	*Reduced*	
Setting: Primary care [36, 44], ED [39], ambulatory care [32], mixed settings [31]	WBC greater or less than the reference for age [39]	Shortness of breath [39]	Fever
		Wheezing [39]	Associated with a raised relative risk of antibiotics in one study based in paediatric primary care offices [36] but not in two others (one set in ambulatory care [32], another with a mix of settings including primary care and ED [31].
	Pleuritic pain [39]		
		Congestion [31]	
	Findings on ear examination [44]	Vomiting in the last day [44]	
Clinical Condition: RTI	C-reactive Protein (CRP) level [39, 44]		
n = 12868	One found a 10 fold increase from normal to be associated with increased odds of antibiotics [39].		
	The second study explored four thresholds: <21, 21–40, 41–60, >60. Of these, only <21 was not associated with increased odds of antibiotics [44].		
	In this study the clinicians knew the CRP test results.		
Setting: General Practice	Ear Discharge [45]		No discrepant findings
Clinical Condition: Otitis Media			
n = 256			

A grey box indicates that no associations were found.

## Discussion

### Summary of results

We found 27 studies fulfilling our inclusion criteria. Nine explored future outcomes and the remainder explored clinical management from the initial consultation. None of the studies were conducted in a home setting and only seven were in primary care.

Two studies investigated future hospitalisation [9, 28]. In one study, moderate-to-severe vomiting, parent reported severe fever or a temperature of ≥37.8°C, intercostal and subcostal recession, and wheeze at the initial contact with primary care were associated with low increases odds of hospitalisation within 30 days [9]. The presence of at least four of seven characteristics (the four symptoms above, in addition to age < 2 years, illness duration of <4 days, and current asthma) was associated with an 11.8% risk of hospitalisation, whilst the presence of zero or only one characteristic reduced risk of hospitalisation in 30 days to 0.3%. In this study the overall risk of hospitalisation was 0.9%. We found a low risk of bias across all but one domains in this study meaning that we consider the results to be reliable. As acknowledged by the authors, the results are most useful in identifying which children are at a low risk of hospitalisation within 30 days of presentation to primary care as 88.2% of those classified as “high risk” did not require hospitalisation.

In the second study [28], increased work of breathing, dehydration and heart rate above 97^th^ centile for each age were associated with low increases of odds of hospitalisation within 24 hours of the initial consultation. We had concerns regarding the study procedures meaning we are unable to make firm conclusions about the relationship between the factors identified and hospitalisation. The overall risk of hospitalisation in this study was 23.6% much higher than that in primary care and, in all probability, the home.

There was little evidence available on prognostic factors association with death. Most factors were not associated with re-consultation or prolonged symptoms. We found two reliable studies indicating that prediction of clinical course based upon symptoms is challenging. In one study, children presenting with parent or carer reported fever and low energy or tiredness, or a clinician recorded cough, were more likely to have worse parent reported symptoms after seven days [7]. No confounding variables were discussed in this study, and we had concerns about the risk of bias from study attrition. In one study, children with a diagnosis of community acquired pneumonia presenting with fever were slightly more likely to re-consult.

One study found that none of 18 CARIFs items reported by parents on the day of the initial consultation or 6 clinician recorded symptoms, signs, and investigations were associated with antibiotic prescription in a follow up period of two weeks [7].

The majority of included studies assessed symptoms, signs, and investigations associated with immediate hospitalisation. Across different settings and conditions, signs of respiratory difficulty such as shortness of breath, a low oxygen saturation, and signs of dehydration were associated with small increases in the odds of hospitalisation. The retrospective nature of this evidence prevents firm conclusions as it is unclear whether any prognostic factors were acknowledged and not recorded, or recorded and not reported.

### Comparison with the literature

The review adds to the literature investigating how undesired consequences of respiratory and other infections can be predicted in primary care. Previous reviews have focused on the predicting serious infection using clinical features at presentation [48, 49], clinical prediction rules [50], laboratory tests [49, 51], and the NICE traffic light system [52]. Much like our study, these reviews find a paucity of studies in a low prevalence, general practice settings. They also find that in low prevalence settings, symptoms and clinical prediction rules have limited rule-in value for serious infection [48–50, 52].

Our study focused on the prediction of poor prognosis. A previous review [11] found little evidence of clinical rules to predict poor prognosis in children presenting with cough and respiratory tract infections, and limited evidence of clinical signs and symptoms to predict pneumonia. Our findings are similar yet our methods are more robust, meaning we can be more confident in the lack of diagnostic rules and the challenges in identifying children at risk of poor outcomes in primary care. Relevant to clinicians, more recent research suggests prediction rules may be more useful for identifying children not requiring antibiotic prescription or referral to secondary care [9]. A recent study found no association between clinician ‘gut feeling’ and poor prognosis [53].

### Strengths and limitations

To our knowledge, this study is the first to systematically review the association between individual symptoms, signs, investigations and prognostic outcomes in children with RTI. The main strength of this review is its size and comprehensive coverage of the literature. Our search was large and we included studies with outcomes on the day of the initial contact in addition to studies with true prognostic outcomes.

However, the review is limited by the paucity and the low quality of the included studies. There was heterogeneity in study design, the included population, outcomes, and prognostic factor definition and measurement. Furthermore, the majority of included studies explored the association between symptoms, signs, and investigations and clinical decision making, but the quality of these clinical decisions was not clear. We also limited our search to high income countries, meaning our results may not be applicable to lower and middle income countries.

Finally, we only analysed individual symptoms, signs, and investigations. We excluded studies investigating groups of symptoms unless individual factors contributing to this were reported separately. Many factors are interlinked, such as respiratory distress and low oxygen saturations, but we were unable to investigate the contribution of individual characteristics when presented as clusters.

### Implications for future research

In order to best explore the hypothesis that symptoms, signs, and clinical investigations can be of use to parents when assessing a child with a respiratory tract infection, future research should consider four design elements. Firstly research should focus on identifying factors that parents can measure to help them decide if clinical input is needed. We identified no studies aiming to identify children who do not need to see a doctor or for whom continued care at home is safe.

Secondly studies should measure symptoms in the ways parents do. Each study in this review took one cross section only to measure the presence of each prognostic factor. Although this is relevant to how clinicians may make a judgement, it may not be relevant to how parents assess illness in their child which includes assessment of change in symptoms over time and with over the counter medications.

Thirdly, studies should be set in the home in order to explore factors of greatest relevance to parents.

Finally, studies should interpret how their results may impact parental decision making. This should focus on whether changes in odds of poor outcomes are large enough that care in the home is no longer safe.

## Conclusions

We found nine studies exploring associations between symptoms, signs, and investigations and prognostic outcomes in children presenting with RTIs to primary care. Two studies provide evidence which may be used to stratify risk of future hospitalisation. One of these was conducted in primary care and we considered it to be of good quality. The other, condition in the ED, was found to be at high risk of confounding and less relevant to GPs and parents. Current evidence is insufficient to enable clinicians to identify children at risk of death, re-consultation, antibiotic prescription, or prolonged or deteriorating symptoms from examination and history at one consultation. This is due to poor quality studies, as well as very few results indicating that this is possible. There were no studies in a home setting, and no studies explicitly aiming to identify clinical features which mean it is safe to continue caring for children in the home. Research asking whether parents can identify children at risk of poor prognosis in the home is needed.

## Supporting information

S1 File (PDF)

S1 Checklist PRISMA 2009 checklist. (DOC)

## Abbreviations

NHS: National Health Service
ED: emergency department
RTI: respiratory tract infection
GP: general practitioner
CARIFS: Canadian Acute Respiratory Illness and Flu Scale
