# Supplementary material for: Predicting poor outcomes in children aged 1–12 with respiratory tract infections: A systematic review
Source: PLoS One. 2021 Apr 19;16(4):e0249533. doi: 10.1371/journal.pone.0249533 (PMC8055026; doi:10.1371/journal.pone.0249533)
Supplement: S1 File — (PDF) [file pone.0249533.s001.pdf]

# ▲	Searches	Results
1	exp child/ or infant/	2148481
2	(infan* or baby or babies or child* or schoolchild* or girl* or boy* or pediatric* or paediatric*).ti,ab.	1994135
3	1 or 2	2916719
4	exp Respiratory Tract Infections/	372165
5	((respiratory or chest) adj3 (infect* or inflam*)).ti,ab.	55981
6	(ARI or ARTI or URTI or LRTI).ti,ab.	5778
7	(pharyngit* or nasopharyngit* or naso-pharyngit* or rhinopharyngit* or rhino-pharyngit* or sinusit* or nasosinusit* or naso-sinusit* or rhinosinusit* or rhino-sinosit* or rhinit* or rhinorrhoea or rhinorrhea or ((runny or running or discharg* or congest* or blocked or stuff* or dripping or runn*) adj2 (nose* or nasal))).ti,ab.	60350
8	((throat* adj3 (sore or pain or inflam* or infect*)) or tonsillit* or laryngit* or rhinolaryngit* or rhino-laryngit* or nasolaryngit* or naso-laryngit* or sinonasal* or sino-nasal*).ti,ab.	20838
9	(croup or pseudocroup or tracheitis or tracheobronchit* or laryngotracheobronchit* or bronchit* or bronchiolit* or pneumon* or pleuropneumon* or bronchopneumon* or pleurisy).ti,ab.	225591
10	(cough or sneez* or common cold).ti,ab.	50324
11	(influenza or flu).ti,ab.	104432
12	(otitis media or aom or ome or earache*).ti,ab.	28335
13	4 or 5 or 6 or 7 or 8 or 9 or 10 or 11 or 12	680650
14	Ambulatory Care/	43060
15	exp Ambulatory Care Facilities/	54992
16	general practice/ or family practice/	74977
17	general practitioners/ or physicians, family/ or physicians, primary care/	27564

18	Primary Health Care/	77672
19	Office Visits/	6942
20	exp Emergency Service, Hospital/	78761
21	Emergency Medical Services/	42768
22	(ambulatory adj3 (care or setting? or facilit* or ward? or department? or service?)).ti,ab.	17562
23	((general or family) adj2 (practi* or physician? or doctor?)).ti,ab.	118793
24	(primary care or primary health care or primary healthcare).ti,ab.	135021
25	(emergency adj3 (care or setting? or facilit* or ward? or department? or service?)).ti,ab.	124088
26	(after hour? or afterhour? or "out of hour?" or ooh).ti,ab.	4836
27	(clinic? or visit?).ti,ab.	503769
28	home.ti,ab.	220379
29	((health* or medical or walk-in or walkin) adj2 (center? or centre?)).ti,ab.	126466
30	(general practi* or primary care or primary health* or family pract*).in,jw.	122523
31	14 or 15 or 16 or 17 or 18 or 19 or 20 or 21 or 22 or 23 or 24 or 25 or 26 or 27 or 28 or 29 or 30	1303774
32	(Inappropriate Prescribing/ or Drug Prescriptions/) and Anti-Bacterial Agents/	3215
33	((antibiotic? or anti-biotic? or antibacterial? or anti-bacterial? or antimicrobial? or anti-microbial?) adj3 (prescri* or "use" or overuse or overprescri* or usage or consum* or uptake or delay* or demand? or reduc* or discontinu* or stop*)).ti,ab.	54579
34	Practice Patterns, Physicians'/	59976
35	(reconsult* or re-consult* or ((repeat* or "follow up" or further or subsequent*) adj3 (consult* or visit* or appointment))).ti,ab.	28493

36	mortality/ or child mortality/ or infant mortality/ or prognosis/	578051
37	(mortality or death?).ti,ab.	1E+06
38	hospitalization/ or patient admission/	130024
39	(((hospital or patient) adj2 admi*) or hospitali?ation or hospitali?ed).ti,ab.	290101
40	(symptom? adj5 (prolong* or non-resolv* or duration or worsen*)).ti,ab.	28925
41	(infection? adj5 (prolong* or non-resolv* or duration or worsen*)).ti,ab.	10761
42	((symptom? or infection) adj2 (serious or sever*)).ti,ab.	62376
43	32 or 33 or 34 or 35 or 36 or 37 or 38 or 39 or 40 or 41 or 42	2300882
44	3 and 13 and 31 and 43	7424
45	Pregnancy/ or Pregnant Women/ or (pregnant* or pregnancy or antenatal or prenatal).ti.	909363
46	44 not 45	7164
47	exp HIV/ or exp HIV INFECTIONS/ or exp Malaria/ or exp Tuberculosis/ or Cystic Fibrosis/ or (hiv or human immunodeficien* virus or malaria or tuberculosis or tb or cystic fibrosis).ti.	662444
48	46 not 47	6754
49	Developing Countries/	74872
50	(Africa or Caribbean or West Indies or South America or Latin America or Central America).hw,ti,ab,cp.	202677

51	(Afghanistan or Albania or Algeria or Angola or American Samoa or Armenia or Armenian or Azerbaijan or Bangladesh or Benin or Byelarus or Byelorussian or Belarus or Belorussian or Belorussia or Belize or Bhutan or Bolivia or Bosnia or Herzegovina or Hercegovina or Botswana or Brazil or Brasil or Bulgaria or Burkina Faso or Burkina Fasso or Upper Volta or Burundi or Urundi or Cambodia or Khmer Republic or Kampuchea or Cameroon or Cameroons or Cameron or Camerons or Cape Verde or Central African Republic or Chad or China or Colombia or Comoros or Comoro Islands or Comores or Mayotte or Congo or Zaire or Costa Rica or Cote d'Ivoire or Ivory Coast or Cuba or Djibouti or French Somaliland or Dominica or Dominican Republic or East Timor or East Timur or Timor Leste or Ecuador or Egypt or United Arab Republic or El Salvador or Eritrea or Ethiopia or Fiji or Gabon or Gabonese Republic or Gambia or Gaza or Georgia Republic or Georgian Republic or Ghana or Gold Coast or Grenada or Guatemala or Guinea or Guinea-Bisau or Guam or Guiana or Guyana or Haiti or Honduras or India or Maldives or Indonesia or Iran or Iraq or Jamaica or Jordan or Kazakhstan or Kazakh or Kenya or Kiribati or Korea or Kosovo or Kyrgyzstan or Kirghizia or Kyrgyz Republic or Kirghiz or Kirgizstan or Lao PDR or Laos or Lebanon or Lesotho or Basutoland or Liberia or Libya or Macedonia or Madagascar or Malagasy Republic or Malaysia or Malaya or Malay or Sabah or Sarawak or Malawi or Nyasaland or Mali or Marshall Islands or Mauritania or Mauritius or Agalega Islands or Mexico or Micronesia or Middle East or Moldova or Moldovia or Moldovian or Mongolia or Montenegro or Morocco or Ifni or Mozambique or Myanmar or Myanma or Burma or Namibia or Nepal or Netherlands Antilles or Nicaragua or Niger or Nigeria or Pakistan or Palau or Palestine or Panama or Papua New Guinea or Paraguay or Peru or Philippines or Philipines or Phillipines or Phillippines or Romania or Rumania or Roumania or Rwanda or Ruanda or Saint Lucia or St Lucia or Saint Vincent or St Vincent or Grenadines or Samoa or Samoan Islands or	2664010

	Navigator Island or Navigator Islands or Sao Tome or Senegal or Serbia or Sierra Leone or Sri Lanka or Ceylon or Solomon Islands or Somalia or Sudan or Suriname or Surinam or Swaziland or Syria or Principe or South Sudan or Tajikistan or Tadzhikistan or Tadjikistan or Tadzhik or Tanzania or Thailand or Timor-Leste or Togo or Togolese Republic or Tonga or Tunisia or Turkey or Turkmenistan or Turkmen or Tuvalu or Uganda or Ukraine or Uzbekistan or Uzbek or Vanuatu or New Hebrides or Vietnam or Viet Nam or West Bank or Yemen or Zambia or Zimbabwe or Rhodesia).hw,ti,ab,cp.	

52	((developing or less* developed or under developed or underdeveloped or middle income or low* income or underserved or under served or deprived or poor*) adj (countr* or nation? or state? or population? or world)).ti,ab.	102236
53	((developing or less* developed or under developed or underdeveloped or middle income or low* income) adj (economy or economies)).ti,ab.	565
54	(low* adj (gdp or gnp or gross domestic or gross national)).ti,ab.	247
55	(low adj3 middle adj3 countr*).ti,ab.	16973
56	(lmic or lmics or third world or lami countr*).ti,ab.	7787
57	transitional countr*.ti,ab.	160
58	49 or 50 or 51 or 52 or 53 or 54 or 55 or 56 or 57	2846481
59	48 not 58	5014
60	(comment or editorial or letter or news or "review").pt. or case report.ti.	4902863
61	59 not 60	4415
62	limit 61 to english language	4025
63	limit 48 to "reviews (maximizes specificity)"	243
64	limit 63 to english language	240
65	62 or 64	4215

Table S1 – Medline Search strategy

**Study**	**Symptoms Included**
Ahmed 2010	Sore throat, cough, fever*, congestion**
Alzaharani 2018	“Fever”
Ambroggio 2018[a]	Fever (38+)*, hypoxia (oxygen saturation 92 or below), tachycardia, tachypnoea,
Bender 2009	Historical: respiratory distress, nasal congestion, emesis, cough, fever, poor oral intake, abdominal pain, diarrhoea.

	Clinical evidence of respiratory distress*, wheeze on examination, fever at time of examination, cough at time of examination, otitis media at time of examination.<br>Radiographic evidence of focal pneumonia*<br>Laboratory confirmed influenza A, laboratory influenza B*<br>CRP, White blood cell count, alanine aminotransferase, erythrocyte sedimentation rate, and aspartate aminotransferase.
Blacklock 2011	**Parent reported**: cough, difficult or laboured breathing, pale colour, wheeze, rash or skin sports, fever or high temperature, irritable or miserable, refusing food/feeds, runny nose, vomiting, nausea, tummy pain, cold hands/feet, sore throat, headache, aches all over, ear pain, confused, hurts to look at lights, pain in legs or arms, neck painful or stiff.<br>**Triage nurse assessment**: cough, respiratory distress*, pallor*, wheeze, dehydration, rash, non-blanching rash<br>Viral signs: RR > APLS, temperature ≥38oC, temperature ≥39oC*, Saturation <94%*, Pulse >APLS, pulse >90th percentile for age and temperature
Brown 2003	Oxygen saturations
Butler 2005	Corzya, raised temperature, pharyngitis, enlarged lymph nodes, malaise<br><br>CARIFs items: poor appetite, not sleeping well, irritable/cranky/fussy, feels unwell, low energy/tired, not playing well, crying more than usual, needing extra care, clinginess, headache, sore throat, muscle aches or pains, fever, cough, nasal congestion or runny nose, vomiting, not interested in what's going on, unable to get out of bed.
Ciofi Degli Atti 2006	Fever*

Dalziel 2013	**Current illness**: dyspnoea, apnoea, headache, nausea/vomiting, diarrhoea, increased/purulent sputum, seizures, generalised weakness, dizziness, irritable/drowsy, collapse/syncope/dizziness, wheezing, chest pain, rhinorrhoea.<br>**Physical exam:** fever >38.9, respiratory rate (linear and quadratic), heart rate (linear, quadratic, cubic), on oxygen or oxygen saturation <93%*, chest retractions*, accessory muscle use, crepitations/rales, wheeze/rhonchi, prolonged CRT/shocked, altered mental status, signs of dehydration*<br>**Laboratory and radiographic investigation**: abnormal chest radiograph, lobar pneumonia, bronchopneumonia, haemoglobin <10g/dl, total leukocyte count >15,000/ul, pH <7.3, bicarbonate <21nmol/L, urea >20 mg/dl, glucose >200 mg/dl, platelet count (three thresholds).
Dubnov-Raz 2011	**Symptoms**: fever, nasal discharge, cough, shortness of breath*, sore throat, vomiting, diarrhoea, myalgia, abdominal pain, rash, non-specific symptoms<br>**Physical examination:** abnormal auscultation*, abnormal heart findings, abnormal abdominal findings, degree of fever, heart rate, oxygen saturation, abnormal chest roentgenogram*
Florin 2020	ANC, CRP, WBC, Procalcitonin
Garcia 2015	Chills**, congestion**, cough, diarrhoea, dizziness, dyspnoea*, fatigue*, fever**, headache**, myalgia*, rhinorrhoea, sore throat, tachycardia*, vomiting, wheezing
Gotta 2017	CRP*, wheezing**, WBC*, age, temperature* dyspnoea**, reduced breathing sound, crackles, bronchial breathing, late inspiratory crackles, heart rate normal for age, respiratory rate normal for age, pleural rub*
Hay 2003	Moderate or severe illness, abnormal chest signs, fever, tachypnoea, dehydration

Hay 2016	**Carer-reported**: general symptoms ≤3 days, severity of illness ≥7 days, breathing quickly, wheezing (at any point), vomiting (at any point in illness), change in crying (at any point in the illness). **Carer reported symptoms in the last 24 hours**: moderate-to-severe vomiting*, severe fever*, severe disturbed sleep, breathing quickly, moderate-to-severe wheezing in chest, severe reduction in eating, moderate-to-severe reduction in urine passed, severe dry cough, moderate-to-severe reduced fluid intake, recent illness deterioration, productive or wet cough, barking or croupy cough, blocked runny nose, chills, shivering, low energy, fatigue, lethargy. **General clinical examination**: irritable or drowsy, pallor, nasal flaring, grunting, temperature ≥37.8*, oxygen saturation ≤95%, severity of illness ≥4/10, clinicians gut feeling that something is wrong, bronical breathing, inflamed pharynx or tonsils, age-adjusted tachycardia, capillary refill time, stridor. **Chest examination**: tachypnoea, recession*, wheeze*, crackles or crepitations
Millar 2007	Fever ≥ 38oC, decreased oral intake*, drooling, trismus, dehydration*, uvular deviation*, airway compromise, voice changes, leukocyte count >$15x10^9$/L, neck radiograph abnormal, intravenous antibiotics, fine-needle aspiration, incision and drainage, quinsy tonsillectomy, observed drooling*
Pailhouse 2015	Oxford saturations ≤94%*, respiratory rate ≥60*, food intake of less than 50% of the usual amount*, clinical deterioration*, digestive problems,
Parker 2009	Temperature, respiratory rate ≥60 at baseline*, accessory muscle score of the RDAI at baseline ≥6/9*, oxygen saturation ≤92% at baseline*, decreased hydration*
Pruikkonen 2014	Oxygen saturation**

Rebnord 2017	Temperature measured, respiratory rate, CRP (various thresholds*), Oxygen saturation (various threshold*), Parents assessment of sickness, vomiting in last day*, earache in the last 24 hours, dsypnoea in the last 24 hours, findings on ear examination*, signs on auscultation*
Smith 2010	Ear discharge*
Venkatesan 2015	Severe respiratory distress, increase respiratory rate, oxygen saturation ≤92% or breathing air or on oxygen, respiratory exhaustion or apnoeic episode, evidence of severe clinical dehydration or clinical shock, altered conscious level, causing other clinical concern to their own GP team.
Walsh 2004	**Information from the history:** difficulty breathing*, cough, fever at home by parental report, decreased feeding, wheeze, vomiting, diarrhoea, decreased activity level, nasal discharge, apnoeic episodes or cyanotic spells.<br>**Physical examination:** cyanosis (none, peripheral, central), dehydration* (none, mild, severe,), level of consciousness (alert, drowsy, lethargic), increase work of breathing (more than mild intercostal or subcostal retractions, nasal flaring), grunting, auscultatory findings, tachycardia about 9th centile for age*
Voets 2006	Respiratory frequency (45/minute threshold)*, oxygen saturation (95% threshold)*
Wensaas 2018	Carer-reported: general symptoms ≤3 days, severity of illness ≥7 days, breathing quickly, wheezing (at any point), vomiting (at any point in illness), change in crying (at any point in the illness).<br>Carer reported symptoms in the last 24 hours: moderate-to-severe vomiting, severe fever, severe disturbed sleep, breathing quickly, moderate-to-severe wheezing in chest, severe reduction in eating, moderate-to-severe reduction in urine passed, severe dry cough, moderate-to-severe reduced fluid intake, recent illness deterioration,

	productive or wet cough, barking or croupy cough, blocked runny nose, chills, shivering, low energy, fatigue, lethargy.<br>General clinical examination: irritable or drowsy, pallor, nasal flaring, grunting, temperature ≥37.8, oxygen saturation ≤95%, severity of illness ≥4/10, clinicians gut feeling that something is wrong, bronical breathing, inflamed pharynx or tonsils, age-adjusted tachycardia, capillary refill time, stridor.<br>Chest examination: tachypnoea, recession, wheeze, crackles or crepitations
Yang 2017	Chest wall retraction (none, mild, moderate, severe), stridor (none, with agitation, at rest, no cyanosis, alertness), air entry (none, decreased, markedly decreased), fever, croup history
Yusuf 2012	Fever, increased work of breathing, difficulty feeding, pulse oximetry <93%*, respiratory rate >60 breaths/min

Table S2 – All relevant prognostic factors by study. *Denotes a PF with a positive statistical relationship with the outcome (increases odds/liklelihood of outcome)
** Denotes a PF with a negative statistical relationship with the outcome (decreases odds/liklelihood of outcome)
